# 1,3-Bis(hydroxy­meth­yl)benzimidazolin-2-one

**DOI:** 10.1107/S1600536809040963

**Published:** 2009-10-17

**Authors:** H. C. Devarajegowda, V. Madhura, B. S. Palakshamurthy, S. Jeyaseelan, Manohar V. Kulkarni

**Affiliations:** a Department of Physics, Yuvaraja’s College (Constituent College), University of Mysore, Mysore 570 005, Karnataka, India; b Department of Chemistry, Karnatak University, Dharwad 580 003, Karnataka, India

## Abstract

The title compound, C_9_H_10_N_2_O_3_, crystallizes with one and a half mol­ecules in the asymmetric unit, one lying on a general position and the other on a twofold rotation axis. The dihedral angle between the two independent benzimidazole ring systems is 18.96 (5)°. In the crystal, mol­ecules are linked into a three-dimensional network by O—H⋯O hydrogen bonding involving *N*-hydroxy­methyl and carbonyl groups, and C—H⋯O hydrogen bonds.

## Related literature

For general background to 2-benzimidazolones, see: Raghu *et al.* (2005 ▶); Porret & Hebermeier (1974 ▶); Habermeier (1976 ▶); Trask-Morrel *et al.* (1988 ▶); Hammach *et al.* (2006 ▶); Bansal *et al.* (1981 ▶). For related structures, see: Anklekar & Kulkarni (1995 ▶); Schwiebert *et al.* (1996 ▶). For the synthesis, see: Zinner & Spangenberg (1958 ▶). 
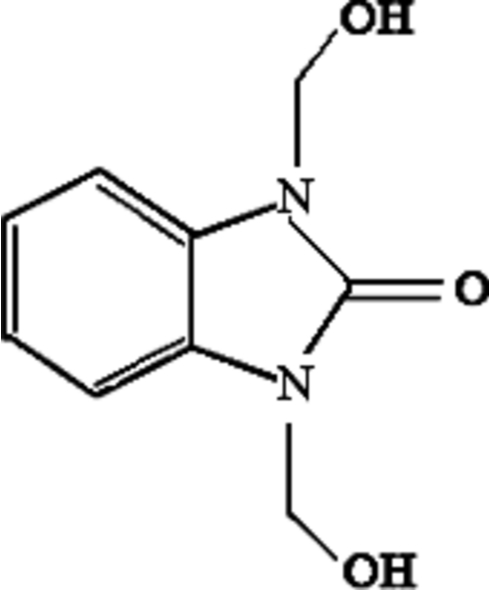


## Experimental

### 

#### Crystal data

C_9_H_10_N_2_O_3_
*M* *_r_* = 194.19
Monoclinic, 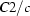

*a* = 13.5515 (14) Å
*b* = 11.0848 (12) Å
*c* = 17.6253 (19) Å
β = 94.216 (2)°
*V* = 2640.4 (5) Å^3^
*Z* = 12
Mo *K*α radiation
μ = 0.11 mm^−1^
*T* = 273 K
0.22 × 0.20 × 0.10 mm

#### Data collection

Bruker SMART CCD area-detector diffractometer
Absorption correction: multi-scan (*SADABS*; Sheldrick, 2004 ▶) *T*_min_ = 0.976, *T*_max_ = 0.986
13521 measured reflections
2684 independent reflections
2395 reflections with *I* > 2σ(*I*)
*R*_int_ = 0.025

#### Refinement

*R*[*F* ^2^ > 2σ(*F* ^2^)] = 0.040
*wR*(*F* ^2^) = 0.110
*S* = 1.10
2684 reflections
191 parameters
H-atom parameters constrained
Δρ_max_ = 0.16 e Å^−3^
Δρ_min_ = −0.24 e Å^−3^

### 

Data collection: *SMART* (Bruker, 2001 ▶); cell refinement: *SAINT* (Bruker, 2001 ▶); data reduction: *SAINT*; program(s) used to solve structure: *SHELXS97* (Sheldrick, 2008 ▶); program(s) used to refine structure: *SHELXL97* (Sheldrick, 2008 ▶); molecular graphics: *ORTEP-3* (Farrugia, 1997 ▶); software used to prepare material for publication: *SHELXL97*.

## Supplementary Material

Crystal structure: contains datablocks global, I. DOI: 10.1107/S1600536809040963/ci2925sup1.cif

Structure factors: contains datablocks I. DOI: 10.1107/S1600536809040963/ci2925Isup2.hkl

Additional supplementary materials:  crystallographic information; 3D view; checkCIF report

## Figures and Tables

**Table 1:** Hydrogen-bond geometry (Å, °)

*D*—H⋯*A*	*D*—H	H⋯*A*	*D*⋯*A*	*D*—H⋯*A*
O1—H1*A*⋯O4	0.82	1.99	2.8003 (16)	169
O2—H2*A*⋯O5^i^	0.82	1.93	2.7503 (18)	176
O5—H5*A*⋯O3^ii^	0.82	1.84	2.6551 (17)	175
C3—H3⋯O2^iii^	0.93	2.58	3.489 (2)	164
C14—H14*B*⋯O1^ii^	0.97	2.54	3.364 (2)	143

Symmetry codes: (i) 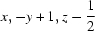; (ii) 
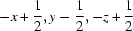
; (iii) 
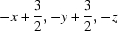
.

**Fig. 1.**
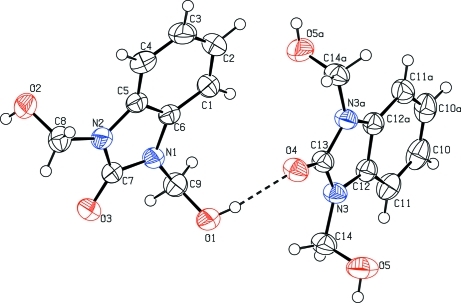
The asymmetric unit of the title compound. Displacement ellipsoids are drawn at the 50% probability level.

**Fig. 2.**
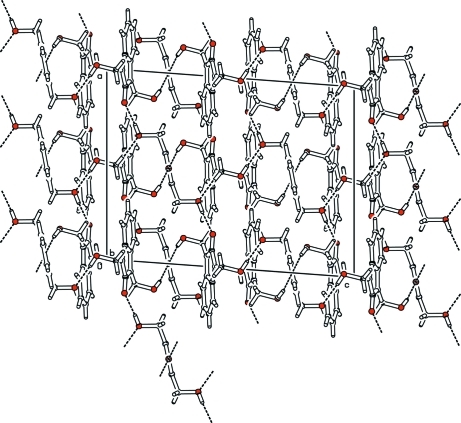
Packing of the molecules viewed down the b axis. Hydrogen bonds are shown as dashed lines.

## supplementary crystallographic information

### Comment

1,3-Bishydroxyalkylated benzimidazolones are an important class of functionalized benzimidazoles which have been found to be useful as polymer intermediates (Raghu *et al.*, 2005; Porret *et al.*, 1974), fire retardants (Habermeier, 1976), and in curing process in textile industry (Trask-Morrel *et al.*, 1988). Solid state chemistry of hydroxy methylated benzimidazole derivatives leading to thermal extrusion of formaldehyde has been reported (Anklekar *et al.*, 1995) by our group. Design and synthesis of benzimidazolone p38 MAP kinase inhibitors (Hammach *et al.*, 2006) is based on the analysis of their crystal structure data. Benzimidazolones have been reported to crystallize as hydrogen bonded molecular tapes (Schwiebert *et al.*, 1996) which has been used to engineer structures of organic solids. In view of the therapeutic importance of aromatic and hetero aromatic compounds (Bansal *et al.*, 1981) containing *N*-hydroxymethyl group, we report here the crystal structure of title compound.

The asymmetric unit of the title compound contains one and a half molecules, one lying on a general position and and the other on a twofold rotation axis (Fig.1). Atoms O4 and C13 lie on the twofold rotation axis. The two independent benzimidazole ring systems form a dihedral angle of 18.96 (5)°. There are no intramolecular hydrogen bonding between *N*-hydroxymethyl and carbonyl groups.

In the crystal, O—H···O hydrogen bonding involving *N*-hydroxymethyl and carbonyl groups results in the formation of three-dimensional network (Fig.2). In addition, C—H···O hydrogen bonds (Table 1) are observed. This type of intermolecular association is similar to that observed in the structure of benzimidazolone (Schwiebert *et al.*, 1996).

### Experimental

The title compound was prepared by following a literature method (Zinner *et al.*, 1958). A mixture of 2-hydroxy benzimidazole (13.4 g, 0.01 *M*) and 37% formalin (30 ml, 1*M*) was refluxed for 30 minutes in presence of 100 ml water. The solid product formed was filtered and single crystals were grown by slow evaporation in water (yield 92%, m.p. 433 K). Spectral data IRν_CO_ = 1700 cm^-1^, ν_OH_ = 3300 cm^-1^. ^1^H NMR -(CDCl_3_+DMSO-d6)δ p.p.m. - 5.3(4*H*, d, CH_2_) appeared as singlet on D_2_O exchange, 6.2(2*H*, t, OH) vanished on D_2_O exchange, 7.4–7.8 (4H, m, Ar-H). Mass m/*z* = 134 (100%).

### Refinement

H atoms were positioned at calculated positions [O-H = 0.82 Å and C-H = 0.93–0.97 Å] and refined using a riding model with *U*_iso_(H) = 1.2*U*_eq_(C) and 1.5*U*_eq_(O).

### Crystal data

**Table:**

C_9_H_10_N_2_O_3_	*F*(000) = 1224
*M**_r_* = 194.19	*D*_x_ = 1.465 Mg m^−^^3^
Monoclinic, *C*2/*c*	Melting point: 433 K
Hall symbol: -C 2yc	Mo *K*α radiation, λ = 0.71073 Å
*a* = 13.5515 (14) Å	Cell parameters from 2684 reflections
*b* = 11.0848 (12) Å	θ = 2.3–26.4°
*c* = 17.6253 (19) Å	µ = 0.11 mm^−^^1^
β = 94.216 (2)°	*T* = 273 K
*V* = 2640.4 (5) Å^3^	Plate, white
*Z* = 12	0.22 × 0.20 × 0.10 mm

### Data collection

**Table:**

Bruker SMART CCD area-detector diffractometer	2684 independent reflections
Radiation source: fine-focus sealed tube	2395 reflections with *I* > 2σ(*I*)
graphite	*R*_int_ = 0.025
ω and φ scans	θ_max_ = 26.4°, θ_min_ = 2.3°
Absorption correction: multi-scan (*SADABS*; Sheldrick, 2004)	*h* = −16→16
*T*_min_ = 0.976, *T*_max_ = 0.986	*k* = −13→13
13521 measured reflections	*l* = −22→22

### Refinement

**Table:**

Refinement on *F*^2^	Primary atom site location: structure-invariant direct methods
Least-squares matrix: full	Secondary atom site location: difference Fourier map
*R*[*F*^2^ > 2σ(*F*^2^)] = 0.040	Hydrogen site location: inferred from neighbouring sites
*wR*(*F*^2^) = 0.110	H-atom parameters constrained
*S* = 1.10	*w* = 1/[σ^2^(*F*_o_^2^) + (0.0486*P*)^2^ + 1.5821*P*] where *P* = (*F*_o_^2^ + 2*F*_c_^2^)/3
2684 reflections	(Δ/σ)_max_ = 0.001
191 parameters	Δρ_max_ = 0.16 e Å^−^^3^
0 restraints	Δρ_min_ = −0.24 e Å^−^^3^

### Special details

**Table:**

Geometry. All e.s.d.'s (except the e.s.d. in the dihedral angle between two l.s. planes) are estimated using the full covariance matrix. The cell e.s.d.'s are taken into account individually in the estimation of e.s.d.'s in distances, angles and torsion angles; correlations between e.s.d.'s in cell parameters are only used when they are defined by crystal symmetry. An approximate (isotropic) treatment of cell e.s.d.'s is used for estimating e.s.d.'s involving l.s. planes.
Refinement. Refinement of *F*^2^ against ALL reflections. The weighted *R*-factor *wR* and goodness of fit *S* are based on *F*^2^, conventional *R*-factors *R* are based on *F*, with *F* set to zero for negative *F*^2^. The threshold expression of *F*^2^ > σ(*F*^2^) is used only for calculating *R*-factors(gt) *etc*. and is not relevant to the choice of reflections for refinement. *R*-factors based on *F*^2^ are statistically about twice as large as those based on *F*, and *R*-factors based on ALL data will be even larger.

### Fractional atomic coordinates and isotropic or equivalent isotropic displacement parameters (Å^2^)

**Table:**

	*x*	*y*	*z*	*U*_iso_*/*U*_eq_	
O1	0.36019 (9)	0.44213 (11)	0.19000 (7)	0.0555 (3)	
H1A	0.4009	0.3984	0.2131	0.083*	
O2	0.51268 (9)	0.85515 (11)	−0.04107 (7)	0.0560 (3)	
H2A	0.4597	0.8565	−0.0667	0.084*	
O3	0.32846 (8)	0.69395 (11)	0.07135 (7)	0.0525 (3)	
N1	0.44553 (9)	0.54147 (11)	0.09507 (7)	0.0390 (3)	
N2	0.49663 (9)	0.72131 (11)	0.06132 (7)	0.0420 (3)	
C1	0.61458 (12)	0.44272 (15)	0.11034 (9)	0.0469 (4)	
H1	0.5932	0.3665	0.1238	0.056*	
C2	0.71420 (13)	0.46756 (18)	0.10526 (10)	0.0568 (5)	
H2	0.7604	0.4063	0.1146	0.068*	
C3	0.74639 (13)	0.5819 (2)	0.08647 (10)	0.0582 (5)	
H3	0.8138	0.5963	0.0847	0.070*	
C4	0.67978 (12)	0.67501 (17)	0.07028 (9)	0.0499 (4)	
H4	0.7012	0.7516	0.0576	0.060*	
C5	0.58057 (11)	0.64950 (13)	0.07380 (8)	0.0388 (3)	
C6	0.54853 (11)	0.53545 (13)	0.09463 (8)	0.0372 (3)	
C7	0.41401 (11)	0.65604 (14)	0.07545 (8)	0.0398 (3)	
C8	0.49544 (14)	0.84582 (14)	0.03582 (10)	0.0511 (4)	
H8A	0.4317	0.8813	0.0440	0.061*	
H8B	0.5458	0.8909	0.0658	0.061*	
C9	0.37880 (12)	0.44495 (14)	0.11304 (9)	0.0456 (4)	
H9A	0.3166	0.4549	0.0827	0.055*	
H9B	0.4072	0.3684	0.0993	0.055*	
O4	0.5000	0.27510 (13)	0.2500	0.0467 (4)	
O5	0.33811 (9)	0.12882 (13)	0.36950 (7)	0.0628 (4)	
H5A	0.2848	0.1462	0.3858	0.094*	
N3	0.42148 (9)	0.09011 (11)	0.26254 (7)	0.0396 (3)	
C10	0.45046 (15)	−0.24348 (15)	0.25741 (9)	0.0553 (5)	
H10	0.4179	−0.3167	0.2617	0.066*	
C11	0.39872 (13)	−0.13699 (15)	0.26601 (9)	0.0487 (4)	
H11	0.3325	−0.1370	0.2765	0.058*	
C12	0.45049 (11)	−0.03066 (13)	0.25817 (8)	0.0385 (3)	
C13	0.5000	0.16353 (19)	0.2500	0.0379 (4)	
C14	0.32941 (11)	0.13248 (16)	0.28957 (9)	0.0468 (4)	
H14A	0.3162	0.2143	0.2722	0.056*	
H14B	0.2752	0.0814	0.2701	0.056*	

### Atomic displacement parameters (Å^2^)

**Table:**

	*U*^11^	*U*^22^	*U*^33^	*U*^12^	*U*^13^	*U*^23^
O1	0.0523 (7)	0.0567 (7)	0.0602 (7)	0.0107 (6)	0.0216 (5)	0.0184 (6)
O2	0.0488 (7)	0.0629 (8)	0.0571 (7)	0.0001 (6)	0.0090 (5)	0.0226 (6)
O3	0.0415 (6)	0.0568 (7)	0.0603 (7)	0.0122 (5)	0.0109 (5)	0.0124 (5)
N1	0.0370 (6)	0.0371 (6)	0.0436 (7)	0.0016 (5)	0.0077 (5)	0.0042 (5)
N2	0.0454 (7)	0.0366 (7)	0.0451 (7)	0.0036 (5)	0.0122 (5)	0.0077 (5)
C1	0.0514 (9)	0.0423 (8)	0.0477 (9)	0.0097 (7)	0.0092 (7)	0.0043 (7)
C2	0.0463 (10)	0.0675 (12)	0.0574 (10)	0.0190 (8)	0.0094 (8)	0.0066 (9)
C3	0.0382 (9)	0.0814 (13)	0.0561 (10)	0.0022 (8)	0.0110 (7)	0.0020 (9)
C4	0.0479 (9)	0.0547 (10)	0.0486 (9)	−0.0070 (7)	0.0143 (7)	0.0017 (7)
C5	0.0424 (8)	0.0402 (8)	0.0349 (7)	0.0031 (6)	0.0095 (6)	0.0012 (6)
C6	0.0392 (8)	0.0397 (8)	0.0335 (7)	0.0020 (6)	0.0079 (5)	−0.0001 (6)
C7	0.0419 (8)	0.0417 (8)	0.0366 (7)	0.0048 (6)	0.0084 (6)	0.0045 (6)
C8	0.0629 (11)	0.0366 (8)	0.0547 (10)	0.0025 (7)	0.0103 (8)	0.0081 (7)
C9	0.0437 (8)	0.0404 (8)	0.0528 (9)	−0.0040 (7)	0.0052 (7)	0.0027 (7)
O4	0.0426 (8)	0.0344 (8)	0.0635 (10)	0.000	0.0068 (7)	0.000
O5	0.0400 (6)	0.0918 (10)	0.0575 (7)	0.0004 (6)	0.0105 (5)	−0.0143 (7)
N3	0.0328 (6)	0.0377 (7)	0.0490 (7)	−0.0017 (5)	0.0069 (5)	−0.0008 (5)
C10	0.0860 (13)	0.0370 (8)	0.0426 (9)	−0.0116 (8)	0.0021 (8)	0.0014 (7)
C11	0.0562 (10)	0.0459 (9)	0.0439 (8)	−0.0124 (7)	0.0032 (7)	0.0019 (7)
C12	0.0416 (8)	0.0376 (8)	0.0363 (7)	−0.0008 (6)	0.0020 (6)	0.0000 (6)
C13	0.0346 (10)	0.0381 (11)	0.0410 (11)	0.000	0.0025 (8)	0.000
C14	0.0329 (8)	0.0504 (9)	0.0575 (10)	0.0001 (7)	0.0052 (6)	−0.0001 (7)

### Geometric parameters (Å, °)

**Table:**

O1—C9	1.3981 (19)	C5—C6	1.395 (2)
O1—H1A	0.82	C8—H8A	0.97
O2—C8	1.396 (2)	C8—H8B	0.97
O2—H2A	0.82	C9—H9A	0.97
O3—C7	1.2304 (18)	C9—H9B	0.97
N1—C7	1.3759 (19)	O4—C13	1.237 (3)
N1—C6	1.3980 (19)	O5—C14	1.406 (2)
N1—C9	1.4509 (19)	O5—H5A	0.82
N2—C7	1.371 (2)	N3—C13	1.3704 (17)
N2—C5	1.3925 (19)	N3—C12	1.3990 (19)
N2—C8	1.451 (2)	N3—C14	1.4462 (19)
C1—C6	1.378 (2)	C10—C10^i^	1.387 (4)
C1—C2	1.387 (2)	C10—C11	1.387 (3)
C1—H1	0.93	C11—C12	1.384 (2)
C2—C3	1.388 (3)	C11—H11	0.93
C2—H2	0.93	C12—C12^i^	1.393 (3)
C3—C4	1.387 (3)	C13—N3^i^	1.3704 (17)
C3—H3	0.93	C14—H14A	0.97
C4—C5	1.380 (2)	C14—H14B	0.97
C4—H4	0.93		
			
C9—O1—H1A	109.4	O2—C8—H8B	109.2
C8—O2—H2A	109.5	N2—C8—H8B	109.2
C7—N1—C6	109.53 (12)	H8A—C8—H8B	107.9
C7—N1—C9	123.27 (13)	O1—C9—N1	112.84 (13)
C6—N1—C9	127.20 (12)	O1—C9—H9A	109.0
C7—N2—C5	109.76 (12)	N1—C9—H9A	109.0
C7—N2—C8	124.58 (14)	O1—C9—H9B	109.0
C5—N2—C8	125.66 (13)	N1—C9—H9B	109.0
C6—C1—C2	117.39 (16)	H9A—C9—H9B	107.8
C6—C1—H1	121.3	C14—O5—H5A	109.5
C2—C1—H1	121.3	C13—N3—C12	109.55 (12)
C1—C2—C3	121.40 (16)	C13—N3—C14	124.00 (13)
C1—C2—H2	119.3	C12—N3—C14	125.57 (13)
C3—C2—H2	119.3	C10^i^—C10—C11	121.66 (10)
C4—C3—C2	121.16 (16)	C10^i^—C10—H10	119.2
C4—C3—H3	119.4	C11—C10—H10	119.2
C2—C3—H3	119.4	C12—C11—C10	116.74 (16)
C5—C4—C3	117.36 (16)	C12—C11—H11	121.6
C5—C4—H4	121.3	C10—C11—H11	121.6
C3—C4—H4	121.3	C11—C12—C12^i^	121.59 (10)
C4—C5—N2	131.54 (15)	C11—C12—N3	131.53 (14)
C4—C5—C6	121.39 (14)	C12^i^—C12—N3	106.88 (8)
N2—C5—C6	107.05 (13)	O4—C13—N3	126.43 (9)
C1—C6—C5	121.26 (14)	O4—C13—N3^i^	126.43 (9)
C1—C6—N1	131.97 (14)	N3—C13—N3^i^	107.14 (18)
C5—C6—N1	106.76 (12)	O5—C14—N3	108.05 (13)
O3—C7—N2	125.99 (14)	O5—C14—H14A	110.1
O3—C7—N1	127.14 (14)	N3—C14—H14A	110.1
N2—C7—N1	106.87 (12)	O5—C14—H14B	110.1
O2—C8—N2	111.86 (14)	N3—C14—H14B	110.1
O2—C8—H8A	109.2	H14A—C14—H14B	108.4
N2—C8—H8A	109.2		
			
C6—C1—C2—C3	1.2 (3)	C8—N2—C7—N1	177.40 (14)
C1—C2—C3—C4	−1.5 (3)	C6—N1—C7—O3	−178.71 (15)
C2—C3—C4—C5	0.1 (3)	C9—N1—C7—O3	1.2 (2)
C3—C4—C5—N2	179.76 (16)	C6—N1—C7—N2	1.10 (16)
C3—C4—C5—C6	1.6 (2)	C9—N1—C7—N2	−178.97 (13)
C7—N2—C5—C4	−177.04 (16)	C7—N2—C8—O2	−105.28 (17)
C8—N2—C5—C4	4.1 (3)	C5—N2—C8—O2	73.5 (2)
C7—N2—C5—C6	1.36 (16)	C7—N1—C9—O1	−88.27 (17)
C8—N2—C5—C6	−177.55 (14)	C6—N1—C9—O1	91.64 (17)
C2—C1—C6—C5	0.5 (2)	C10^i^—C10—C11—C12	0.8 (3)
C2—C1—C6—N1	−179.32 (15)	C10—C11—C12—C12^i^	0.5 (3)
C4—C5—C6—C1	−1.9 (2)	C10—C11—C12—N3	179.38 (15)
N2—C5—C6—C1	179.46 (14)	C13—N3—C12—C11	−179.51 (14)
C4—C5—C6—N1	177.94 (13)	C14—N3—C12—C11	10.9 (3)
N2—C5—C6—N1	−0.65 (16)	C13—N3—C12—C12^i^	−0.50 (18)
C7—N1—C6—C1	179.60 (16)	C14—N3—C12—C12^i^	−170.07 (15)
C9—N1—C6—C1	−0.3 (3)	C12—N3—C13—O4	−179.81 (7)
C7—N1—C6—C5	−0.27 (16)	C14—N3—C13—O4	−10.04 (16)
C9—N1—C6—C5	179.81 (14)	C12—N3—C13—N3^i^	0.19 (7)
C5—N2—C7—O3	178.30 (15)	C14—N3—C13—N3^i^	169.96 (16)
C8—N2—C7—O3	−2.8 (2)	C13—N3—C14—O5	−89.75 (16)
C5—N2—C7—N1	−1.52 (16)	C12—N3—C14—O5	78.38 (18)

Symmetry codes: (i) −*x*+1, *y*, −*z*+1/2.

### Hydrogen-bond geometry (Å, °)

**Table:**

*D*—H···*A*	*D*—H	H···*A*	*D*···*A*	*D*—H···*A*
O1—H1A···O4	0.82	1.99	2.8003 (16)	169
O2—H2A···O5^ii^	0.82	1.93	2.7503 (18)	176
O5—H5A···O3^iii^	0.82	1.84	2.6551 (17)	175
C3—H3···O2^iv^	0.93	2.58	3.489 (2)	164
C14—H14B···O1^iii^	0.97	2.54	3.364 (2)	143

Symmetry codes: (ii) *x*, −*y*+1, *z*−1/2; (iii) −*x*+1/2, *y*−1/2, −*z*+1/2; (iv) −*x*+3/2, −*y*+3/2, −*z*.
